# A Newly Isolated Strain *Lysobacter brunescens* YQ20 and Its Performance on Wool Waste Biodegradation

**DOI:** 10.3389/fmicb.2022.794738

**Published:** 2022-03-14

**Authors:** Qinyuan Ma, Ya`ning Zhang, Xue Zheng, Fang Luan, Ping Han, Xianghe Zhang, Yanmiao Yin, Xiaoxiao Wang, Xiuzhen Gao

**Affiliations:** School of Life Sciences and Medicine, Shandong University of Technology, Zibo, China

**Keywords:** keratin, wool degradation, whole-genome sequencing, keratinase, *Lysobacter*

## Abstract

Wool keratin is difficult to degrade as comparing to feathers because of its tough secondary structure. In order to develop an approach for high-value utilization of wool fiber waste by keratinolytic microorganisms, which is produced from shearing, weaving, and industrial processing of wool, screening of wool-degrading bacterium with high degradation efficiency were performed in this study. To this end, *Lysobacter brunescens* YQ20 was identified and characterized. The optimized conditions for wool degradation were pH 9.0 and 37°C with 20% liquid volume of Erlenmeyer flask. After fermentation, 15 essential amino acids were detected when wool fiber waste was fermented. The total amino acids produced from 1% wool per hour were 13.7 mg/L. The concentration was 8.6-fold higher than that produced by the strain *Stenotrophomonas maltophilia* BBE11-1, which had previously been reported to have the highest wool-degrading capacity. Our study reports the first *Lysobacter* strain that exhibits efficient wool degradation and yields higher concentrations of amino acids than previously reported strains. Whole-genome sequencing indicated that there were 18 keratinase-like genes in the genome of YQ20, which exhibited a long evolutionary distance from those of *Bacillus*. Therefore, *L. brunescens* YQ20 may have applications in the environmentally friendly management of wool waste as fertilizer in agriculture.

## Introduction

In the wool processing industry, wool is processed into a series of products, including cashmere and yarn, which are then used for the manufacturing of various goods, such as clothing items, carpets, and tapestries. However, an abundance of rough wool fibers are left as wastes (Zoccola et al., 2012). A considerable amount of low-grade wool waste is also generated from abattoir processing and farming wool trimming practices.

Wool is mainly made up of two major types of protein, i.e., keratin proteins and keratin-associated proteins (Plowman et al., 2012). The major component of wool is keratin protein, accounting for approximately 95% of wool components (Shavandi et al., 2016). According to their secondary structures, keratins can be divided into α-keratin and β-keratin, which consist of α-helical coils and β-pleated sheets, respectively (Meyers et al., 2008; McKittrick et al., 2012). α-Keratin is more difficult to degrade than β-keratin because α-keratin has more disulfide bonds (Gupta et al., 2013). Wool contains 50%–60% α-keratins and less amounts of β-keratins; therefore, it is highly resistant to degradation compared with feathers from chickens, ducks, and geese (Daroit and Brandelli, 2014).

Currently, most feathers are processed into feather meal using high-pressure methods or milling, yielding a product commonly used as a protein supplement in animal feeds. Waste wool fibers have been used as fertilizer (Górecki and Górecki, 2010). However, this product is environmentally hazardous, contains limited amino acids, and exhibits poor digestibility (Onifade et al., 1998; Brandelli, 2008; Holkar et al., 2018). Digestion by microbial fermentation is an alternative, high-value, environmentally friendly method compared with chemical and physical methods (Nam et al., 2002; Sharma and Devi, 2018). Keratinases are expressed in a variety of microbes, particularly those of the genera *Bacillus* (Suntornsuk et al., 2005; Cedrola et al., 2012) and *Actinomyces* (Jaouadi et al., 2010) and some species of saprophytic and parasitic fungi (Gradišar et al., 2005). However, although most known keratinases can efficiently degrade feathers, they are not highly active against wool (Fang et al., 2013b). Therefore, it is necessary to identify novel keratinases with higher activity and a broader spectrum of substrates to establish an improved method for the enzymatic conversion of wool and waste wool fibers.

Accordingly, in this study, we screened for novel keratinolytic bacteria with high efficiency. We then isolated *L. brunescens* YQ20 and characterized its wool degradation ability under optimized fermentation conditions. Our findings highlighted the potential value of *L. brunescens* YQ20 in the waste management of wool and provided a solid foundation for future analysis of keratin-degrading enzymes in *L. brunescens* YQ20.

## Materials and Methods

### Materials

Unprocessed wool and wool fiber waste used for fermentation were collected from Shandong Jinliyuan Plush Product Co., Ltd. (Yangxin County, Shandong Province, China).

### Isolation of Bacteria From Soil

Soil samples were collected from two sheepfolds from a village in Tanfang town, Weifang City, Shandong Province, China. The samples were kept for enrichment in distilled water with 1% wool and incubated at 37°C with shaking at 220 rpm for 1 week. Then, the bacteria were isolated by serial dilution and spread plate technology. Casein agar plates were used for screening and isolation. The plates were incubated at 37°C for 48 h. The composition of casein agar medium was as follows 10 g/L casein, 5 g/L MgSO_4_·7H_2_O, 5 g/L NaCl, and 15 g/L agar. Colonies with a hydrolysis zone were selected out for fermentation with wool as the sole carbon source. The wool fermentation medium contained 5 g/L NaCl, 0.5 g/L KH_2_PO_4_, 1 g/L K_2_HPO_4_, 1 g/L MgSO_4_·7H_2_O, and 10 g/L wool (natural pH). Isolates that could degrade wool during fermentation were considered as positive producers. Freshly grown single colonies on casein agar plates were inoculated into 20 ml nutrient broth medium (18 g/L, pH 7.4). The culture was incubated at 37°C and 220 rpm for 24 h in an orbital shaker and then stored in glycerol stock (15%, final concentration) at −80°C.

### Identification of the Keratinase-Producing Bacterium by 16S rRNA

The isolate was identified based on morphological analysis and 16S rRNA gene sequencing. Colony polymerase chain reaction was performed for amplification of the 16S rRNA gene with 27F (5′-AGAGTTTGATCCTGGCTGAG-3′) and 1492R (5′-GGCTACCTTGTTACGACTT-3′) as primers. The amplified DNA was purified and sequence by Genewiz (Suzhou, China). The DNA sequence was submitted to BLAST.^1^ A neighbor-joining phylogenetic tree was constructed based on 16S rRNA sequences using MEGA7.

### Inoculum Preparation

Before fermentation, 400 μl glycerol inoculum was transferred into a 250-ml Erlenmeyer flask containing 50 ml nutrient broth medium and incubated at 37°C and 220 rpm for 24 h. After fermentation, 4% of the inoculum was transferred into a 250-ml flask containing 50 ml fermentation medium.

### Growth Determination and Evaluation of Fermentation by YQ20

Fermentation were performed at 37°C and 220 rpm with 1% of wool fiber waste as a substrate. During growth, fermented broth was collected for determination of the production of amino acids and degradation of wool per 12 h.

Degradation of wool fiber waste and production of amino acids were measured to evaluate the fermentation process. Wool degradation was determined as previously described (Ramakrishna Reddy et al., 2017). Production of amino acids was determined as follows. First, the fermentation broth was boiled at 100°C for 10 min and then centrifugated at 10,000 × *g* for 15 min. The supernatant was diluted with distilled water and measured at 280 nm.

### Optimization of Wool Waste Concentration

A series of wool fiber waste concentrations (1%, 2%, 3%, 4%, 5%, 6%, 7%, and 8%) were evaluated to determine the appropriate concentration during fermentation. The other components of the fermentation medium were the same as described above. The fermentation was carried out at 37°C and 220 rpm for 36 h.

### Effects of Temperature, pH, and Oxygen on Wool Waste Degradation

In order to determine the suitable conditions for fermentation, including temperature, pH, and oxygen, single-factor experiments were performed with 6% wool fiber waste as substrate.

For optimization of temperature, the culture medium was incubated separately at 25°C, 30°C, 37°C, or 40°C. For determination of the optimum pH for fermentation, fermentation was carried out at pH 5, 6, 7, 8, 9, or 10. The pH was adjusted using 0.1 N HCl or 0.2 N NaOH. The effects of oxygen on wool waste degradation were studied with different volumes of liquid cultures (25, 50, 75, and 100 ml) in a 250-ml Erlenmeyer flask.

All evaluations of fermentation processes were conducted after 36 h. All experiments were repeated with three parallels. The mean values and standard deviations were recorded.

### Amino Acid Analysis of Fermentation Broth

During fermentation, 6% wool fiber waste was added into a 250-ml Erlenmeyer flask containing 50 ml fermented medium (pH 8.0). After sterilization, fermentation was carried out with 4% inoculum at 37°C and 220 rpm. When OD280 no longer increased, fermentation broth was filtered using Whatman No. 1 filter paper, and the obtained filtrate was then assessed using an amino acid analyzer (L-8900; Hitachi, Japan) for determination of amino acid contents.

### Genome Sequencing and *de novo* Genome Assembly

For Single Molecule Real Time (SMRT) sequencing (Pacific Biosciences, Menlo Park, CA), total DNA from YQ20 was extracted with a bacterial genomic DNA extraction kit (Sigma-Aldrich, United States) according to the manufacturer’s instructions. The DNA quality was detected using Qubit (Thermo Fisher Scientific, Waltham, MA, United States) and a Nanodrop instrument (Thermo Fisher Scientific). Qualified genomic DNA was fragmented with G-tubes (Covaris, MA, United States) and end-repaired to prepare SMRTbell DNA template libraries (PacBio, Menlo Park, CA, United States), according to the manufacturer’s specifications. Fragments with sizes of less than 5 Kb were selected using a Bluepippin system. Library quality was detected by Qubit, and the average fragment size was estimated on a Bioanalyzer 2100 (Agilent, Santa Clara, CA, United States). SMRT sequencing was performed on a Pacific Biosciences RSII sequencer (PacBio) according to standard protocols (MagBead Standard Seq v2 loading, 1 × 180 min movie) using P4-C2 chemistry.

Continuous long reads were obtained from three SMRT sequencing runs. Reads longer than 500 bp with a quality value over 0.75 were merged together into a single dataset. The hierarchical genome-assembly process pipeline (Chin et al., 2013) was used to correct for random errors in the long seed reads (seed length threshold: 6 kb) by aligning shorter reads from the same library against them. The resulting corrected, pre-assembled reads were applied to *de novo* assembly using Celera Assembler with an overlap-layout consensus strategy (Myers et al., 2000). To validate the quality of the assembly and determine the final genome sequence, the Quiver consensus algorithm (Chin et al., 2013) was used.

### Genome Annotation and Bioinformatics Analysis

The Glimmer3 program was used for prediction of putative CDSs (Delcher et al., 2007). Prediction of noncoding RNAs, such as rRNAs, was carried out using rRNAmmer (Lagesen et al., 2007), and tRNAs were identified by tNRAscan (Lowe and Eddy, 1997).

Gene function annotation was based on several complementary approaches, including the National Center for Biotechnology Information (NCBI) Nr database, UniProt/SwissProt, KEGG, GO, COG, pfam, and protein families (V26.0).

To identify genes encoding for enzymes potentially involved in keratin degradation, genome mining was performed using amino acid sequences of known enzymes against the genome of *L. brunescens* YQ20 with BLAST (Altschul et al., 2005). Keratinases included those from *Bacillus tequilensis* Q7 (AKN20219.1; Zaraî Jaouadi et al., 2015), *Brevibacillus brevis* strain US575 (AGO58466.1; Jaouadi et al., 2013), *Bacillus circulans* strain DZ100 (AGN91700.1; Benkiar et al., 2013), *Bacillus pumilus* (ANQ68333.1; Fellahi et al., 2016), *Bacillus amyloliquefaciens* K11 (AKR05134.1; Yang et al., 2016), *Bacillus licheniformis* (AFT92040.1; Liu et al., 2013), *Bacillus licheniformis* MKU3 (AAY82467.1; Radha and Gunasekaran, 2007), *Bacillus pumilus* (ANQ68334.1; Fellahi et al., 2016), *F. pennivorans* (AAK61552.1; Kluskens et al., 2002), *Actinomaduraviridilutea* DZ50 (AMH86070.1; Ben Elhoul et al., 2016), *P. aeruginosa* (ADP00718.1; Sharma and Gupta, 2010), *G. stearothermophilus* AD-11 (AJD77429.1; Gegeckas et al., 2015), *S. maltophilia* BBE11-1 (AGK29593.1; Fang et al., 2014), and *Thermoactinomyces* sp. YT06 (APY18977.1; Wang et al., 2019).

The amino acid sequences of proteins, including known keratinases and proteins from YQ20, were initially aligned using ClustalW. Subsequently, phylogenetic trees were constructed using MEGA7 with the best model. The conserved motifs were identified using the Multiple Expectation Maximization for Motif Elicitation online program suite (Bailey et al., 2009). Catalytic domain analysis was carried out using NCBI Batch CDD.^2^ Visual presentation was realized with the integrating toolkit TBtools (Chen et al., 2018).

### Nucleotide Sequence Accession Numbers

The whole-genome sequencing project was assigned BioProject no. PRJNA592006.

## Results

### Isolation and Identification of *Lysobacter brunescens* YQ20

**Figure 1:**
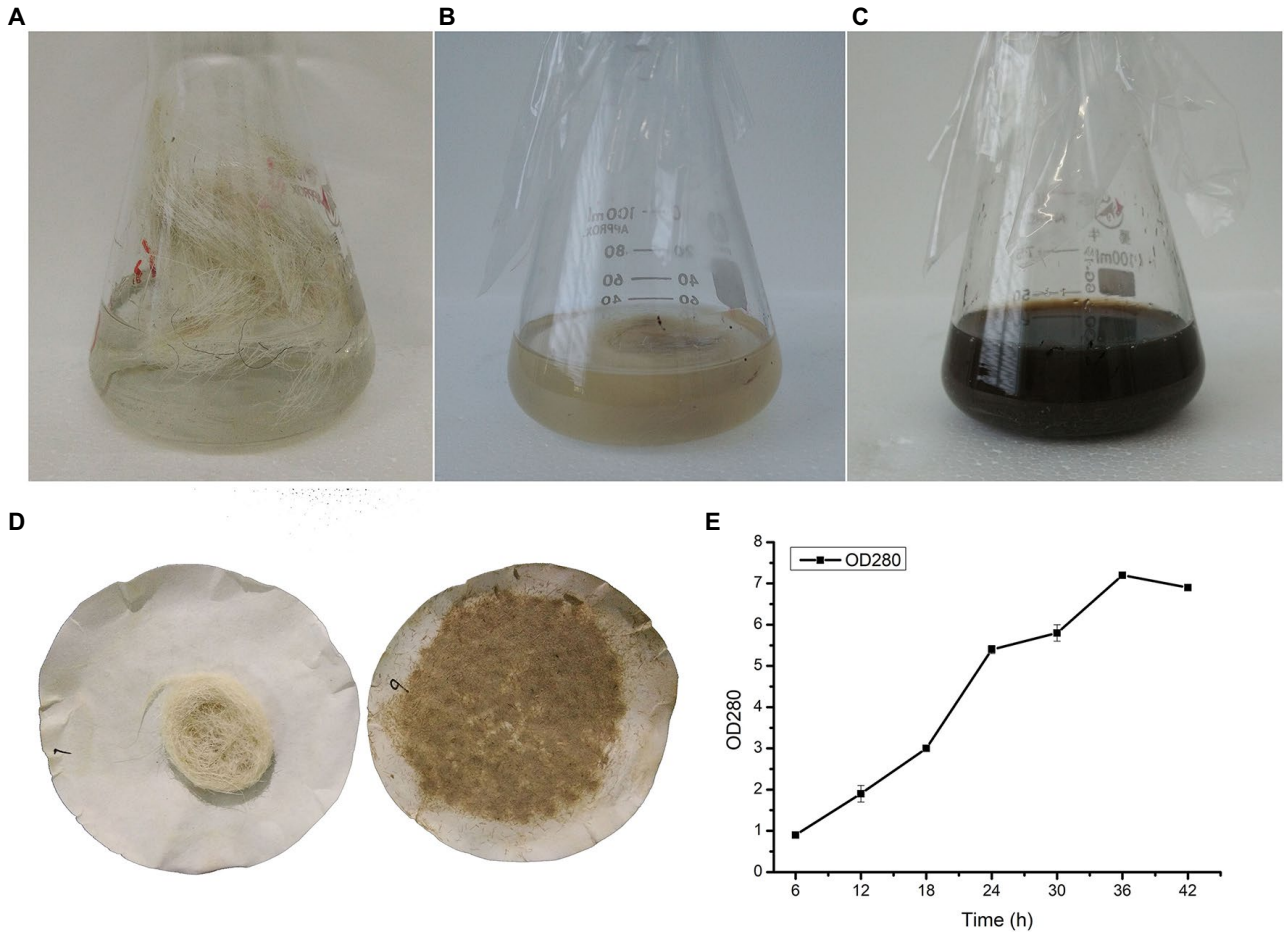
Wool fermentation by *Lysobacter brunescens* YQ20. **(A)** Fermentation medium with unprocessed wool as a substrate, without sterilization. **(B)** Fermentation medium with unprocessed wool as a substrate, sterilization. **(C)** Unprocessed wool after fermentation. **(D)** Filter residues without and with fermentation. **(E)** Time course study on amino acid production during fermentation.

In order to screen out a strain that could degrade wool, we collected soil samples from sheepfolds. Samples were stored for enrichment in distilled water with 1% wool shearing from sheep for 1 week. After cultivation on casein agar plates for 48 h, six colonies produced clear zones, indicating that proteases were secreted. After fermentation with wool as a substrate, there was only one strain that could degrade wool (Figures 1A–C); this strain was designated YQ20. As shown in Figure 1D, after 48 h of fermentation by YQ20, no long wool remained. The time course of wool fermentation by YQ20 is shown in Figure 1E. When fermentation was carried out with 1% wool fiber waste as a substrate, there was a significant increase in amino acids produced until 36 h.

**Figure 2:**
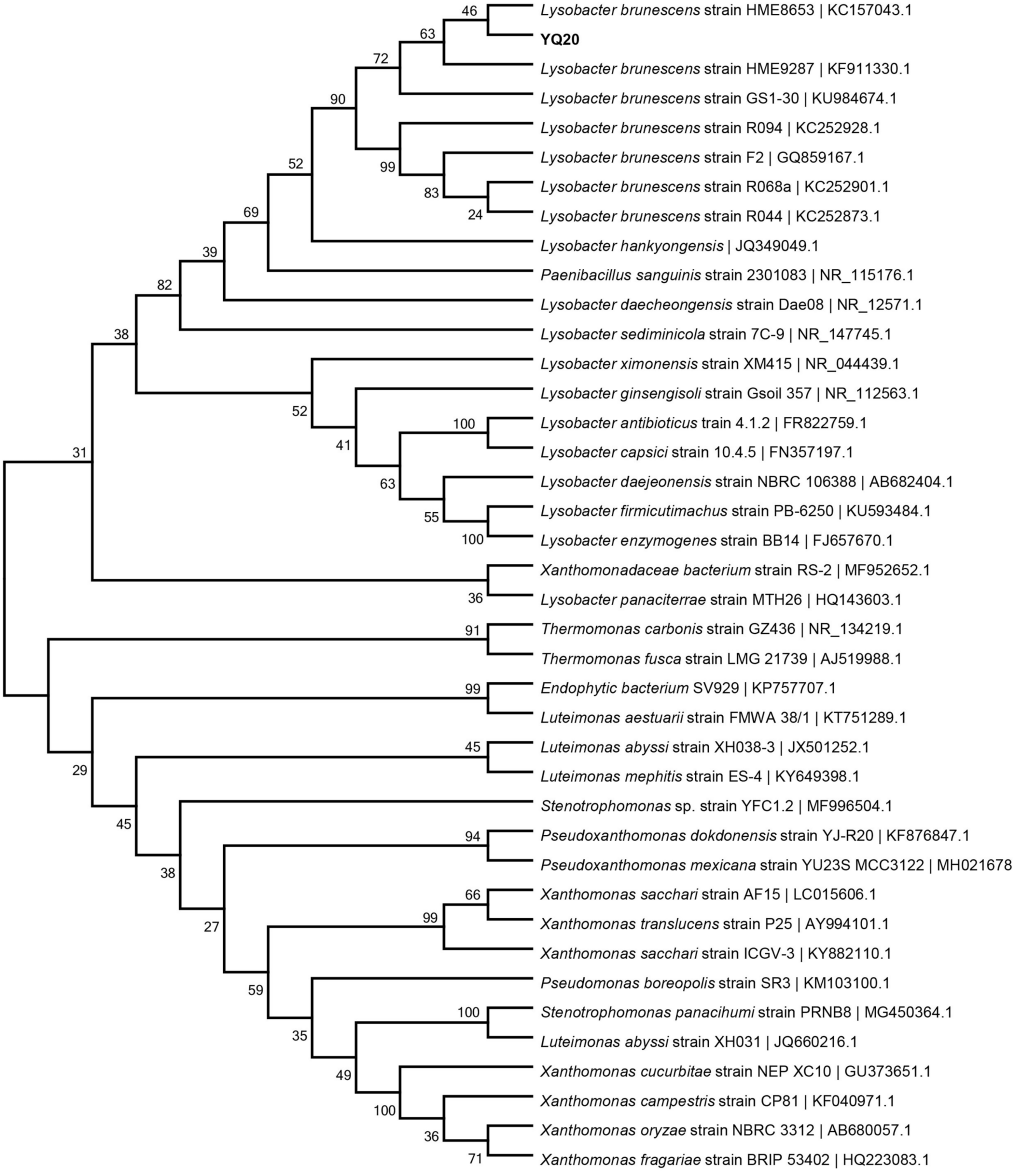
Phylogenetic tree of YQ20. The branching pattern was generated by the neighbor-joining tree method. The GenBank accession numbers of the 16S rRNA nucleotide sequences are indicated in brackets.

The strain YQ20 was then submitted for 16S rRNA sequencing. As shown in Figure 2, YQ20 showed 99% identity with *L. brunescens* strain HME8653. Additionally, the colonies became brown during cultivation, consistent with the known features of *L. brunescens*. Therefore, YQ20 was designated as *L. brunescens* YQ20.

### Optimization of Culture Conditions for the Degradation of Wool Fiber Waste

**Figure 3:**
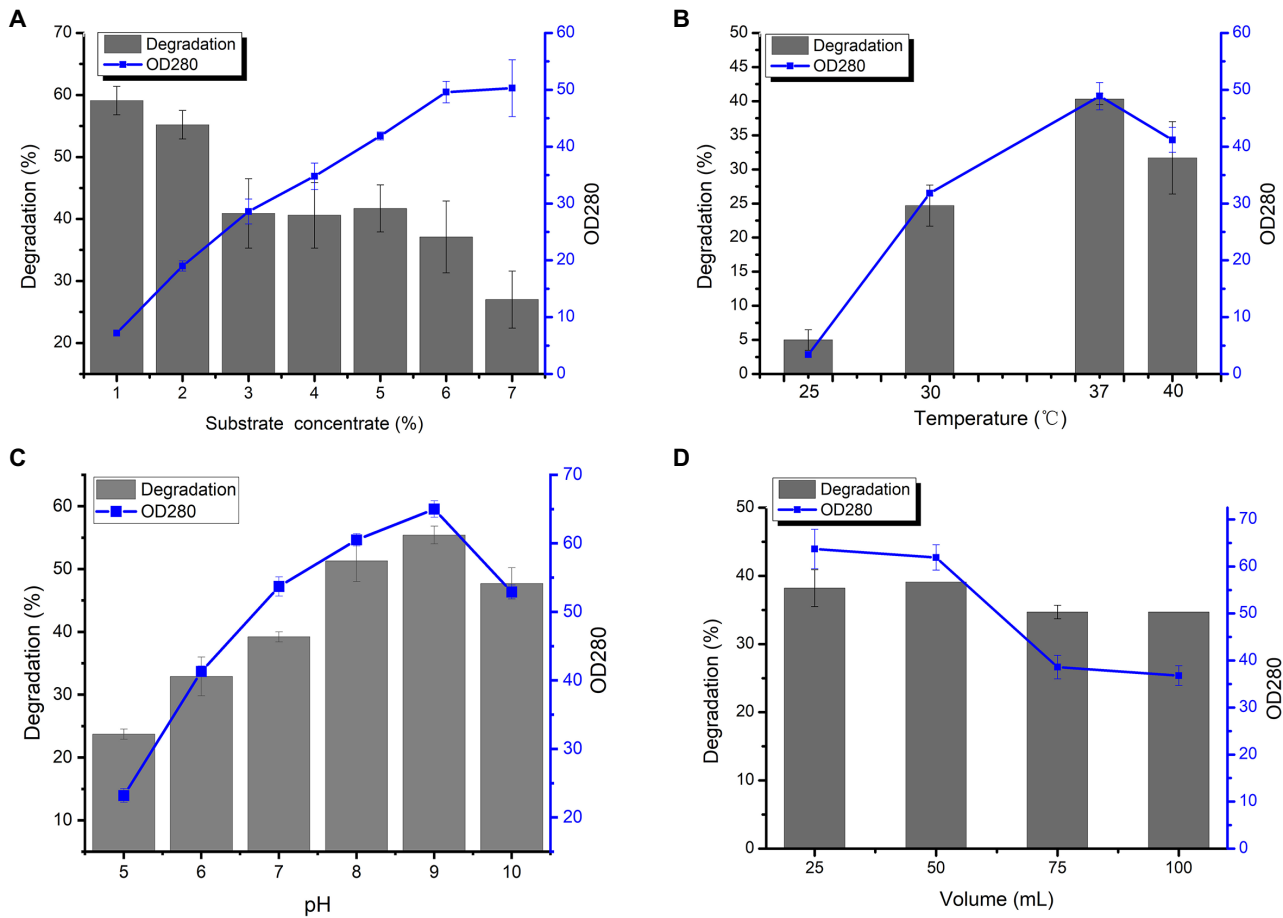
Effects of **(A)** substrate concentration, **(B)** temperature, **(C)** pH, and **(D)** volume of liquid medium on wool fiber waste degradation and amino acid production by *Lysobacter brunescens* YQ20.

From Figure 3A, as the substrate concentration increased, the degradation rate of wool decreased, and the production of amino acids increased linearly until the wool reached 7% of liquid volume. Next, the effects of different fermentation parameters, including pH, temperature, liquid volume, and substrate concentration, were investigated. The highest degradation rate of wool and greatest amount of produced amino acids was found by *L. brunescens* YQ20 after 36 h of fermentation at 37°C and pH 9.0 (Figures 3B,C). In order to investigate the requirement for oxygen during fermentation by *L. brunescens* YQ20, a series of fermentation cultures with different volumes of medium were prepared with the same concentration of wool fiber waste. As shown in Figure 3D, when the liquid in the Erlenmeyer flask was greater than 20% the volume of the flask, the degradation ratio and produced amino acids obviously decreased.

### Determination of Amino Acid Contents in Fermented Broth

**Figure 4:**
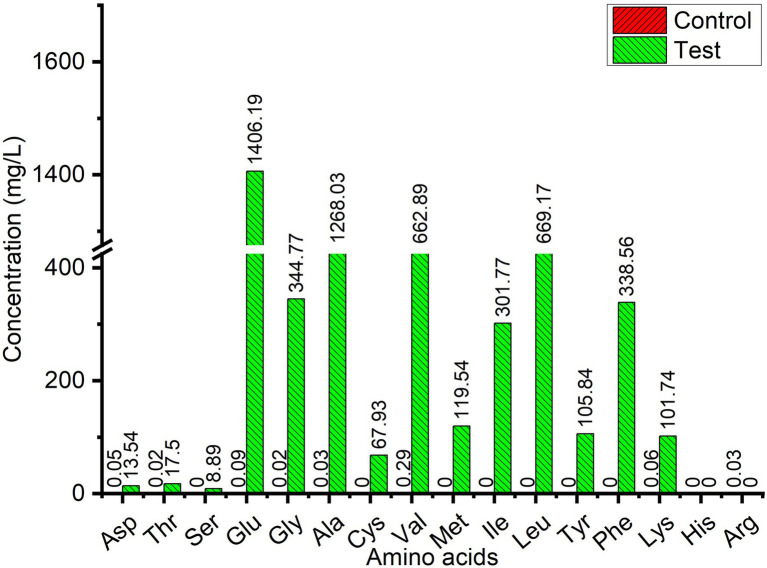
Free amino acid contents in wool fiber waste fermented broth by *Lysobacter brunescens* YQ20. Control, autoclaved feather medium without inoculum; Test, fermentation by *Lysobacter brunescens* YQ20. The control value was too low to measure.

Wool fiber waste from the wool processing industry was used for fermentation by *L. brunescens* YQ20 to analyze the contents of free amino acids in the fermentation broth. From Figure 3D, when the substrate concentration was 7%, the amino acid concentration in the fermentation broth no longer increased, and the degradation rate of wool decreased. Therefore, we chose 6% wool for fermentation to determine the composition and content of amino acids in the fermentation broth. It was found that the OD280 no longer increased after 66 h of fermentation, therefore, fermentation was stopped for amino acid contents analysis. As shown in Figure 4, 15 amino acids were identified when wool waste was degraded, of which Glu was the most abundant (1.4 × 10^3^ mg/L). The total content of free amino acids reached 5.4 × 10^3^ mg/L.

### General Features of the *Lysobacter brunescens* YQ20 Genome

**Figure 5:**
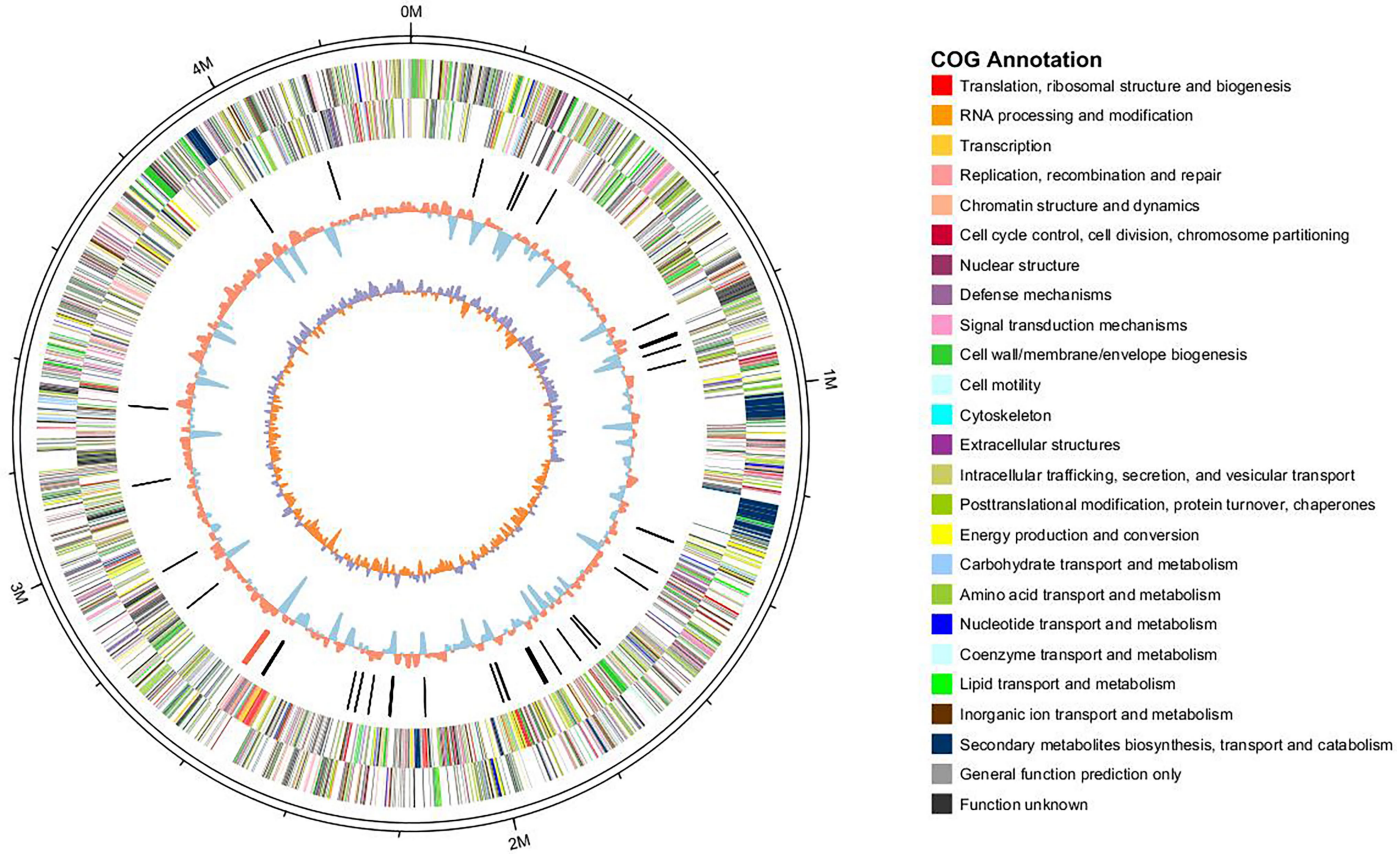
Circle graph of the *Lysobacter brunescens* YQ20 chromosome. Circles are numbered from 1 (outer) to 5 (inner). Circles 1–2, locations of predicted CDSs on + and − strands, respectively; Circle 3, nonprotein coding RNA (ncRNA); Circle 4, %G + C content; Circle 5, GC skew = (G − C)/(G + C).

In order to understand the ability of *L. brunescens* YQ20 to degrade wool, whole-genome sequencing was performed. The circular chromosome of the *L. brunescens* YQ20 genome consisted of 4,358,679 base pairs (bp) with an average G + C content of 68.72%. No plasmids were found during analysis. By prediction using glimmer, 3,932 protein-coding sequences (CDSs) were predicted, accounting for 89.7% of the genome and having lengths ranging from 114 to 14,181 bp. The circle genome was visualized using Circos (version 0.62; Krzywinski et al., 2009). The orientations of all genes are shown in Figure 5 in the direction of replication.

For gene function annotations, different databases, including non-redundant (Nr), Cluster of Orthologous Groups of proteins (COG), Gene Ontology (GO), Kyoto Encyclopedia of Genes and Genomes (KEGG), and SwissProt, were used. We found 3,514 and 2,320 genes with homologs in NR and SwissProt databases, respectively, and 2,589 and 1859 genes could be classified using COG and KEGG, respectively. In total, 3,514 genes could be annotated by these databases, indicating that these genes had been extensively studied. There were 226 genes assigned into biological process, cellular component, and molecular function categories by level 2 GO annotation. The biological process-associated genes were classified into eight categories, among which metabolic process (38.54%), cellular process (25%), and single-organism process (17.71%) were dominant. Among the seven identified cellular component categories, most expressed genes were enriched in cells (31.25%), cell part (31.25%), and macromolecular complex (21.25%). Molecular function-related genes were classified into only four groups, i.e., binding (48%), catalytic activity proteins (32%), structural molecule activity (18%), and transporter activity (one gene).

### Keratinase-Like Gene Candidates and Sequence Analysis

In order to mine possible keratinase genes in the genome of *L. brunescens* YQ20, 14 known keratinases from different genera were used for BLAST search. Finally, 18 proteins were searched out and annotated as subtilase family, which belongs to subtilisin-like serine protease.

**Figure 6:**
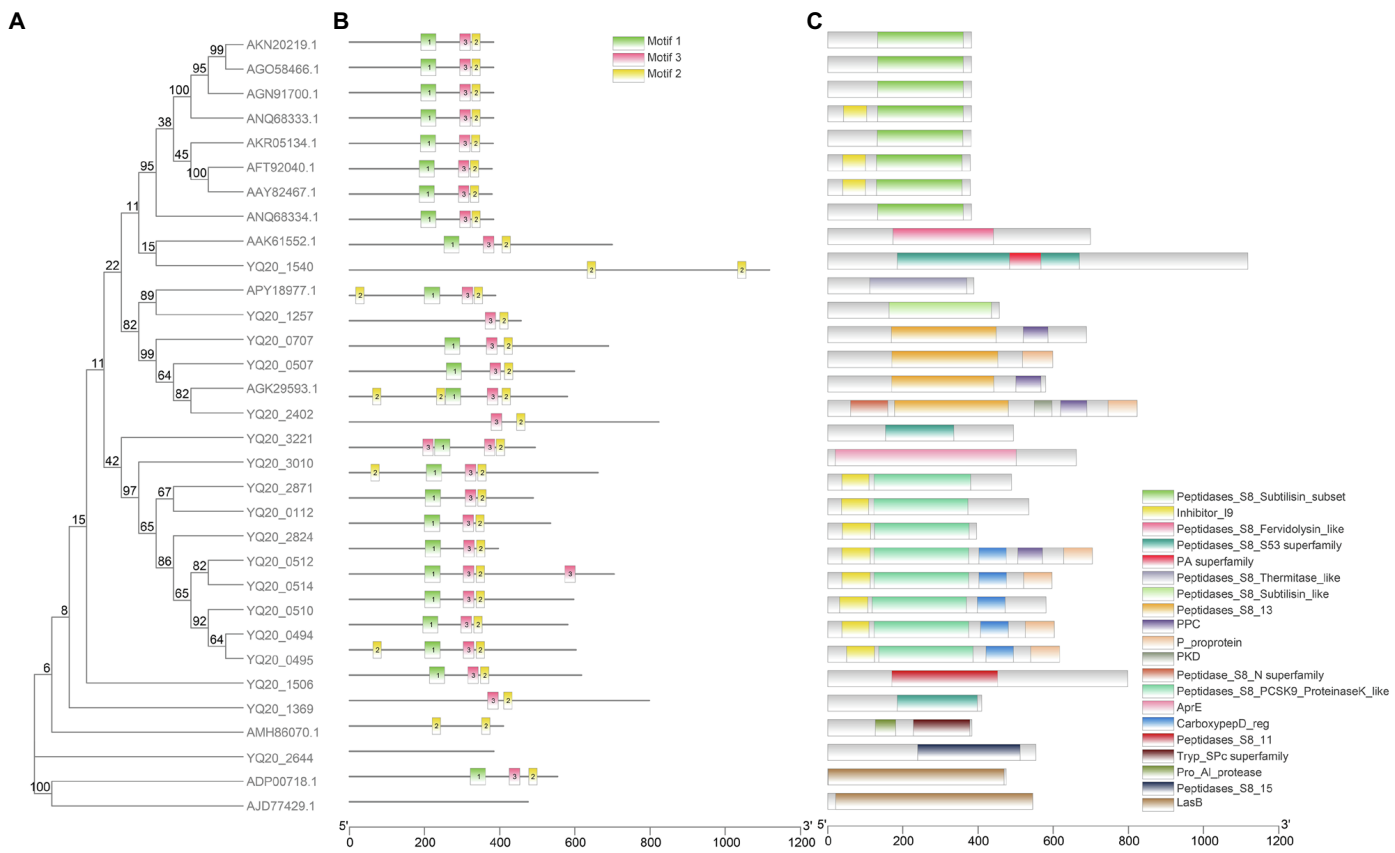
Phylogenetic relationships, conserved protein motifs, and conserved domains of known keratinase and proteins from *Lysobacter brunescens* YQ20 by Blastp. **(A)** Phylogenetic tree. The phylogenetic tree was constructed using the Maximum Likelihood method with 500 bootstrap replications and a WAG model. Keratinases from Bacillus are colored in green. **(B)** Motif composition of all proteins. The motifs are displayed in different colored boxes. **(C)** Conserved domain analysis by NCBI CDD. The protein length can be estimated using the scale bar at the bottom.

Figure 6 shows integration of the results of phylogenetic analysis of amino acids, gene structures, and conserved motifs of known keratinases and proteins from *L. brunescens* YQ20. Notably, keratinases from the genus *Bacillus* aggregated as a clan (Figure 6A), but keratinases from the other genera and serine proteases from *L. brunescens* YQ20 were separate, indicating that these proteins had long evolutionary relationships and differentiation. Based on the phylogenetic tree, the keratinase family showed obvious diversity in sequence. Using the MEME suite, 10 conserved motifs were identified. As shown in Figures 6B,C, keratinases from *Bacillus* in clad I had their own specific motifs and belonged to the S8 subtilisin subset family. Multiple sequence alignment indicated that amino acid sequence similarities between eight keratinases from the genus *Bacillus* remained above 50%. Keratinases from *Fervidobacterium pennivorans* (AAK61552.1), *Thermoactinomyces* sp. YT06 (APY18977.1), and *Stenotrophomonas maltophilia* BBE11-1 (AGK29593.1), which showed approximately 35% similarity to those from *Bacillus*, were distributed in different clads in the phylogenetic tree (Figure 6A), harbored different motifs, and belonged to S8 serine protease. Although keratinases from *Pseudomonas aeruginosa* (ADP00718.1) and *Geobacillus stearothermophilus* AD-11 (AJD77429.1) showed degradation abilities toward keratin, these proteins did not harbor the same conserved motifs as other known keratinases (Figure 6B), and the similarities were lower than 16% for the other keratinases. Additionally, Conserved Domains Database (CDD) analysis indicated that these proteins were Zn-dependent metalloproteases (LasB in Figure 6C).^3^

As shown in Figure 6A, YQ20 proteins clustered in clads II and III, had their own specific motifs, and belonged to different S8 serine protease subfamilies (Figures 6B,C). The similarities between YQ20 keratinase-like proteins and known keratinases were less than 35%, except for those from YQ20_2402, YQ20_0507, and YQ20_0707 (59.6, 49.6, and 48.1%, respectively) and the enzyme from *S. maltophilia*BBE11-1 (AGK29593.1).

## Discussion

### Isolation and Characterization of *Lysobacter brunescens* YQ20 for Wool Waste Degradation

Previous studies have shown that most keratinases are serine proteases (Gradišar et al., 2005). The main industrial producers of serine proteases are strains belonging to the genera *Bacillus* (Jaouadi et al., 2010; Maeda et al., 2011; da Silva, 2018), and *Streptomyces* (Demir et al., 2015). Among all the reported keratinases, two have been reported to be produced by members of the genus *Lysobacter*. One producing strain is *Lysobacter* NCIMB 9497, which expresses a keratinase with 2% powdered keratin as a substrate when cultured for 31 days (Allpress et al., 2002). The other is *Lysobacter* sp. A03, a cold-adapted bacterium (Pereira et al., 2014) that shows maximum proteolytic activity on day 5 when cultivated in feather meal broth containing 1% feather meal. Although the keratinase from *Lysobacter* has been purified and characterized, its degradation ability toward wool had not yet been investigated. Notably, *L. brunescens* YQ20 identified in this study was the first *Lysobacter* strain shown to have high degradation efficiency toward wool. The optimization of fermentation conditions indicated that the keratinase from *L. brunescens* YQ20 had a pH optimum in the alkaline range and a temperature optimum at 37°C, similar to keratinases from other bacteria (Riffel et al., 2003; Lateef et al., 2015; Ramakrishna Reddy et al., 2017). Moreover, the process of degradation required oxygen.

Currently, some wild-type keratinase-producing strains from other genera have been reported to degrade wool directly when fermentation, including some *Bacillus* strains (Gousterova et al., 2005; Hassan et al., 2013; Laba and Rodziewicz, 2014) and *S. maltophilia* BBE11-1 (Fang et al., 2013a,b). However, the genus *Lysobacter* has not been reported to degrade wool. Accordingly, *L. brunescens* YQ20 is the first *Lysobacter* strain shown to degrade wool. *Stenotrophomonas maltophilia* BBE11-1 was a kertin-degrading strain which was isolated from a poultry farm. According to the reports, the purified enzymes form *S. maltophilia* BBE11-1 could degrade native duck feather and wool for different days (Fang et al., 2013a). After fermentation for 48 h in a 30-L fermenter, the strain *S. maltophilia* BBE11-1 was found to degrade 66.7% of native wool. The total amount of amino acids was 153.32 mg/L after 96 h of fermentation with 1% native wool as the substrate; the most abundant essential amino acid was phenylalanine, reaching 92.67 mg/L (Fang et al., 2013b). Prior to our study, this was the strain showing the best wool-degrading capacity. However, in our study, after fermentation for 66 h with 6% wool waste from the wool processing industry as substrate, *L. brunescens* YQ20 yielded a total amino acid content of 5.43 × 10^3^ mg/L, indicating that *L. brunescens* YQ20 produced 13.7 mg/L free amino acids from 1% wool fermentation per hour, which was 8.6-fold higher than that of *S. maltophilia* BBE11-1. In the fermentation broth, the concentration of phenylalanine was 338.56 mg/L, and glutamate exhibited the highest concentration of any amino acid (1.4 × 10^3^ mg/L). Taken together, these results indicated that *L. brunescens* YQ20 exhibited excellent degradation ability toward wool.

Species of the genus *Lysobacter* are frequently found in natural soils (Seccareccia et al., 2015) and produce volatile organic compounds and antimicrobial compounds (de Boer et al., 2019), which can inhibit spore germination and mycelial growth of several phytopathogens, promote plant growth, and induce plant resistance (Vlassi et al., 2020). In addition, species of the genus *Lysobacter* secrete a variety of lytic enzymes, including proteases, glucanases, chitinases, and cellulases (Seccareccia et al., 2015). Therefore, *L. brunescens* YQ20 may be a good candidate for the bio-utilization of wool fiber waste from the wool processing industry to high-value fertilizer in agriculture or additives as animal feed. More studies are needed to scale up and optimize the fermentation process.

### Analysis of Keratinolytic Genes Based on the Whole-Genome Sequence of *Lysobacter brunescens* YQ20

Keratinases are a class of keratinolytic proteases, most of which are serine-type proteases according to sequence analysis (Su et al., 2020). The proteins searched out by Blastp with known keratinases were annotated as subtilisin-like serine proteases, consistent with our previous knowledge. For the genome of *Bacillus* sp. JM7, there was only one gene which was predicted to encode a subtilisin-like serine protease after genome sequencing, and this protein was verified experimentally as a keratinase (Jin et al., 2019). According to a review by Vivek (Salwan and Sharma, 2019), eight bacteria, including *Actinomadura viridilutea* (accession no.: NZ_PVNI01000001.1), *Bacillus pumilus* ATCC 7061 (ac-cession no.: NZ_ABRX01000001-NZ_ABRX01000016), *Burkholderia pseudomallei* (ac-cession no.: CH899711-CH899721, DS981341-DS981409), *Caldanaerobacter subterraneus* KB-1 (accession no.: NZ_AXDC01000001-NZ_XDC01000102), *Dermatophilus congolensis* DSM44180 (accession no.: KE386981-KE386981), *P. flavipulchra* (accession no.: JH650741-JH650756), *P. aeruginosa* (accession no.: KK111587-KK111849), and *Thermus aquaticus* (accession no.: ABVK02000001:ABVK02000022), express 11 serine proteases. Moreover, species of the genus *Lysobacter* produce a series of exoenzymes, including proteases, to utilize food sources (Christensen and Cook, 1978). Thus, *L. brunescens* YQ20 has a natural advantage for keratin degradation. Analysis of the gene structure and conserved motifs indicated that keratinases show obvious differences in sequences, and keratinase-like proteins from YQ20 had a long evolutionary distance from the keratinase of *Bacillus*. These results indicated that the keratinases in *L. brunescens* YQ20 may be distinct from the known keratinases of *Bacillus*. Accordingly, additional studies including proteomic analysis, transcriptomic analysis, and experiments of keratinases expression will be carried out to identify the keratinase genes in YQ20.

Few keratinases have been shown to have high activity against wool. In this study, we aimed to improve the bio-utilization of wool fiber waste from the wool processing industry by isolating a keratinolytic bacterium with efficient wool degradation activity. We obtained the first strain from the genus *Lysobacter* to have wool degradation activity. After characterization, *L. brunescens* YQ20 showed improved degradation ability toward wool compared with *S. maltophilia* BBE11-1, which had previously been identified as the bacterium with the highest wool-degrading capacity. In future studies, large-scale fermentation should be carried out to optimize the industrial process. Moreover, genome sequencing analysis indicated that YQ20 may also harbor novel keratinases, and future studies are needed to verify these findings and elucidate the degradation mechanisms of this strain.

## Data Availability Statement

The datasets presented in this study can be found in online repositories. The names of the repository/repositories and accession number(s) can be found at: https://www.ncbi.nlm.nih.gov/genbank/, no. PRJNA592006.

## Author Contributions

XG: conceptualization, writing—review and editing, and funding acquisition. QM: methodology, writing—original draft preparation, and project administration. YZ, XZe, PH, FL, XZa, and YY: investigation and validation. All authors contributed to the article and approved the submitted version.

## Funding

This project was supported by the Natural Science Foundation of Shandong Province (grant nos. ZR2020MC053 and ZR202102180372).

## Conflict of Interest

The authors declare that the research was conducted in the absence of any commercial or financial relationships that could be construed as a potential conflict of interest.

## Publisher’s Note

All claims expressed in this article are solely those of the authors and do not necessarily represent those of their affiliated organizations, or those of the publisher, the editors and the reviewers. Any product that may be evaluated in this article, or claim that may be made by its manufacturer, is not guaranteed or endorsed by the publisher.

## Footnotes

^1^ https://blast.ncbi.nlm.nih.gov/Blast.cgi

^2^ https://www.ncbi.nlm.nih.gov/Structure/bwrpsb/bwrpsb.cgi

^3^ https://www.ncbi.nlm.nih.gov/cdd
